# Supplementary material for: The Systems Biology Research Tool: evolvable open-source software
Source: BMC Syst Biol. 2008 Jun 29;2:55. doi: 10.1186/1752-0509-2-55 (PMC2446383; doi:10.1186/1752-0509-2-55)
Supplement: Additional file 1 — SBRT Archive. An archive of the current version of the Systems Biology Research Tool. [file 1752-0509-2-55-S1.zip › sbrt-1.4.0/doc/developers_guide/api/sbrt/shell/text/class-use/AppInputFileLineFormat.html]

Uses of Interface sbrt.shell.text.AppInputFileLineFormat


|  |  |  |
| --- | --- | --- |
| **Overview** \| **Package** \| **Class** \| **Use** \| **Tree** \| **Deprecated** \| **Index** \| **Help** | |  |
| PREV   NEXT | **FRAMES**    **NO FRAMES**     **All Classes** |  |


---


## **Uses of Interface sbrt.shell.text.AppInputFileLineFormat**

| Packages that use AppInputFileLineFormat | |
| --- | --- |
| **sbrt.shell.io** | Provides classes and interfaces for reading and writing files. |
| **sbrt.shell.text** | Provides classes and interfaces for defining formats for basic objects. |

Uses of AppInputFileLineFormat in sbrt.shell.io

Constructors in sbrt.shell.io with parameters of type AppInputFileLineFormat

`ProcessInputFile(java.lang.String fileName,
AppInputFileLineFormat lineFormat)`
  
          Constructs a new process input file from the
provided arguments.

Uses of AppInputFileLineFormat in sbrt.shell.text

| Classes in sbrt.shell.text that implement AppInputFileLineFormat | |
| --- | --- |
| `class` | `AppInputFileLineFormatVersion1`             This class is a concrete implementation of `AppInputFileLineFormat`. |

---


|  |  |  |
| --- | --- | --- |
| **Overview** \| **Package** \| **Class** \| **Use** \| **Tree** \| **Deprecated** \| **Index** \| **Help** | |  |
| PREV   NEXT | **FRAMES**    **NO FRAMES**     **All Classes** |  |


---
